# Antepartum Drug Dependence and Pregnancy- or Birth-related Complications: A Cross-sectional Study of 19 Million Inpatients

**DOI:** 10.7759/cureus.6117

**Published:** 2019-11-10

**Authors:** Naveed Ahmad, Chris A Robert, Alekhya Jampa, Sahar Ashraf, Rikinkumar S Patel

**Affiliations:** 1 Psychiatry, University of Texas, Houston, USA; 2 Obstetrics & Gynecology, Sunrise Hospital, Pune, IND; 3 Obstetrics and Gynecology, Jawaharlal Nehru Medical College, Belgaum, IND; 4 Psychiatry, Mayhill Hospital, Denton, USA; 5 Psychiatry, Griffin Memorial Hospital, Norman, USA

**Keywords:** pregnancy, antepartum, outcomes, psychiatric disorder, substance use disorders, substance abuse, comorbidities, hospitalizated patients

## Abstract

Objective

To evaluate the demographic characteristics, hospitalization outcomes [severity, length of stay (LOS), and total expense], and comorbidities in pregnant patients with antepartum drug dependence (ADD).

Methods

We used the national inpatient sample (NIS) and included 19,170,561 female patients (age: 12-40 years) hospitalized for pregnancy- or birth-related complications and grouped by co-diagnosis of ADD. We used descriptive statistics and Pearson’s chi-square test for categorical data and independent sample T-test for the continuous data to measure the differences in demographic and hospital outcomes. A logistic regression model was used to evaluate the odds ratio (OR) for medical and psychiatric comorbidities.

Results

The hospitalizations with ADD declined initially, from 2010 to 2011, followed by an increase of 50% from 2011 to 2014. White pregnant females (77.5%), and those from low-income families (<25^th^ percentile, 37.1 %) had comorbid ADD. Among medical comorbidities, iron-deficiency anemia was the most prevalent condition in pregnant inpatients (12.0% in ADD vs. 9.2% in non-ADD) followed by obesity and hypertension. Depression (12.9%) was the most prevalent psychiatric comorbidity in ADD inpatients followed by comorbid psychosis (three-fold higher odds). Among patients with substance use disorder (SUD), opioid abuse was the most prevalent one (67.3%) followed by cannabis (11.2%), cocaine (5.7%), amphetamine (4.0%), and alcohol (2.4%). Half of the pregnant inpatients with ADD had moderate severity of illness due to pregnancy or birth-related complications with four-fold higher odds [95% confidence interval (CI): 3.67-8.88]. They also had a higher LOS with a mean difference of 0.88 days (95% CI: 0.904-0.865) and had incurred higher total charges, by USD 3,797 (95% CI: 3,927-3,666), per inpatient admission for pregnancy- or birth-related complications compared to non-ADD inpatients

Conclusion

ADD is associated with the worsening of severity of illness in pregnancy- or birth-related complications and requires acute inpatient care that leads to increased healthcare-related economic burden. The integration of SUD services with primary or maternal care is required to improve outcomes in at-risk women in the reproductive age group.

## Introduction

Substance use disorders (SUD) in pregnancy is widespread throughout the US. Among pregnant women, more than 15.9% have been reported to smoke cigarettes, 5.9% use illicit drugs, while 8.5% use alcohol [1]. Besides cannabis, cocaine and amphetamines are used by a significant proportion of pregnant patients and more than half of them use multiple substances [1]. Drug dependence during pregnancy is associated with terrible outcomes. Antenatal alcohol use results in miscarriage, low birth weight, and congenital anomalies [1,2]. Cigarette smoking leads to ectopic pregnancy, increased infant mortality rate, intrauterine growth retardation, and abruptio placenta [2-4]. Antenatal cocaine/methamphetamine use causes premature rupture of membrane, small for gestational age (SGA) newborns, and placental abruption [2,5]. Cannabis use during pregnancy results in reduced executive functioning and attention span in newborns [6]. Opioid use during pregnancy leads to dreadful complications including respiratory depression, microcephaly, and sudden infant death syndrome [2,7]. A nationwide inpatient study found that one-third of the patients with antepartum mental disorders hospitalized for pregnancy-related complications abused drugs [8].

Multiple pathways that could play an important role in the causation of addiction behavior have been postulated, and disturbed metabolism of dopamine, oxytocin, and glucocorticoids are widely studied [9]. The dopamine pathway, through the mesocortical limbic system in the brain, is involved in the reward and motivation, while it regulates voluntary movement and habit formation through the nigrostriatal dopamine system [10,11]. Secondly, oxytocin is synthesized in the magnocellular neurons and supraoptic nucleus [9]. It is important in the shaping of social behavior, attachment formation, and managing stress. Lastly, amygdala neurons synthesize corticotropin-releasing factor (CRF), which controls steroids through the hypothalamic-pituitary-adrenal axis. It is postulated that early-life experience affects the programming of these pathways and could result in a defective response to life stressors later on [1]. Moreover, the stress of pregnancy could be an additional factor. Bodily changes during pregnancy, younger age, unintended pregnancy, lower socioeconomic status, and lack of knowledge of adverse outcomes of substances used are key contributing factors [1].

Given the magnitude of the problem, it is important to be aware of the epidemiology of SUD during the antepartum period in pregnant patients. We conducted a nationwide study of hospitalizations for pregnancy- or birth-related complications and evaluated the differences in demographic characteristics, medical and psychiatric comorbidities including SUD, and hospital outcomes [severity of illness, length of stay (LOS), and total expense] between inpatients with co-diagnosis of antepartum drug dependence (ADD) versus those without.

## Materials and methods

Data source

A retrospective cohort study was conducted using the healthcare cost and utilization project's (HCUP) national inpatient sample (NIS) data from January 2010 to December 2014 [12]. The NIS database is most commonly utilized to evaluate patterns in demographics and hospital outcomes. It is the largest inpatient database in the US and it covers 4,411 hospitals across 45 states [12]. To protect the privacy of patients, physicians, and hospitals, the identifiers were de-identified [12]. Thus, we did not require the permission of the institutional review board to conduct a study on publicly available de-identified inpatient data. 

Inclusion criteria

We included female patients (age: 12-40 years) with a principal diagnosis of pregnancy- or birth-related complications based on the clinical classification software (CCS) codes [13]. The study population (n = 19,170,561) were further grouped based on co-diagnosis of ADD (n = 67,180). The ICD-9 diagnosis codes used to identify ADD were 648.31 or 648.33.

Variables of interest 

Demographic variables evaluated in this study were age, race, and median household income. [13] To measure the differences in hospital outcomes in inpatients based on co-diagnosis of antepartum mental disorders, the following variables were included: severity of illness, number of chronic conditions, LOS, and total charges incurred [13]. In the NIS, LOS is defined as the number of nights the patient remained in the hospital for the principal diagnosis. Total charges during hospitalization do not include professional fees and non-covered charges [13].

Comorbidities are considered coexisting conditions, and using ICD-9 for alcohol, tobacco, cannabis, opioid, cocaine, amphetamine, and barbiturate use disorders, depression, psychoses, deficiency anemias, diabetes, hypertension, hypothyroidism, and obesity were identified in the discharge diagnosis from DX2 to DX25 in the NIS [13].

Statistical analyses

We used descriptive statistics and Pearson's chi-square test for categorical data and independent sample T-test for the continuous data to measure the differences in demographic characteristics and hospital outcomes. We applied the discharge weight [13], which is provided in the NIS, to attain national representation of the inpatient population. Differences in comorbidities were quantified using chi-square tests. We used a binomial logistic regression model to evaluate the odds ratio (OR) for the severity of illness, medical and psychiatric comorbidities, and SUD. All regression models were adjusted for demographic confounders. Data were analyzed using IBM SPSS Statistics for Windows, version 25.0 (IBM, Armonk, NY). A p-value of <0.01 was deemed statistically significant.

## Results

**Figure 1:**
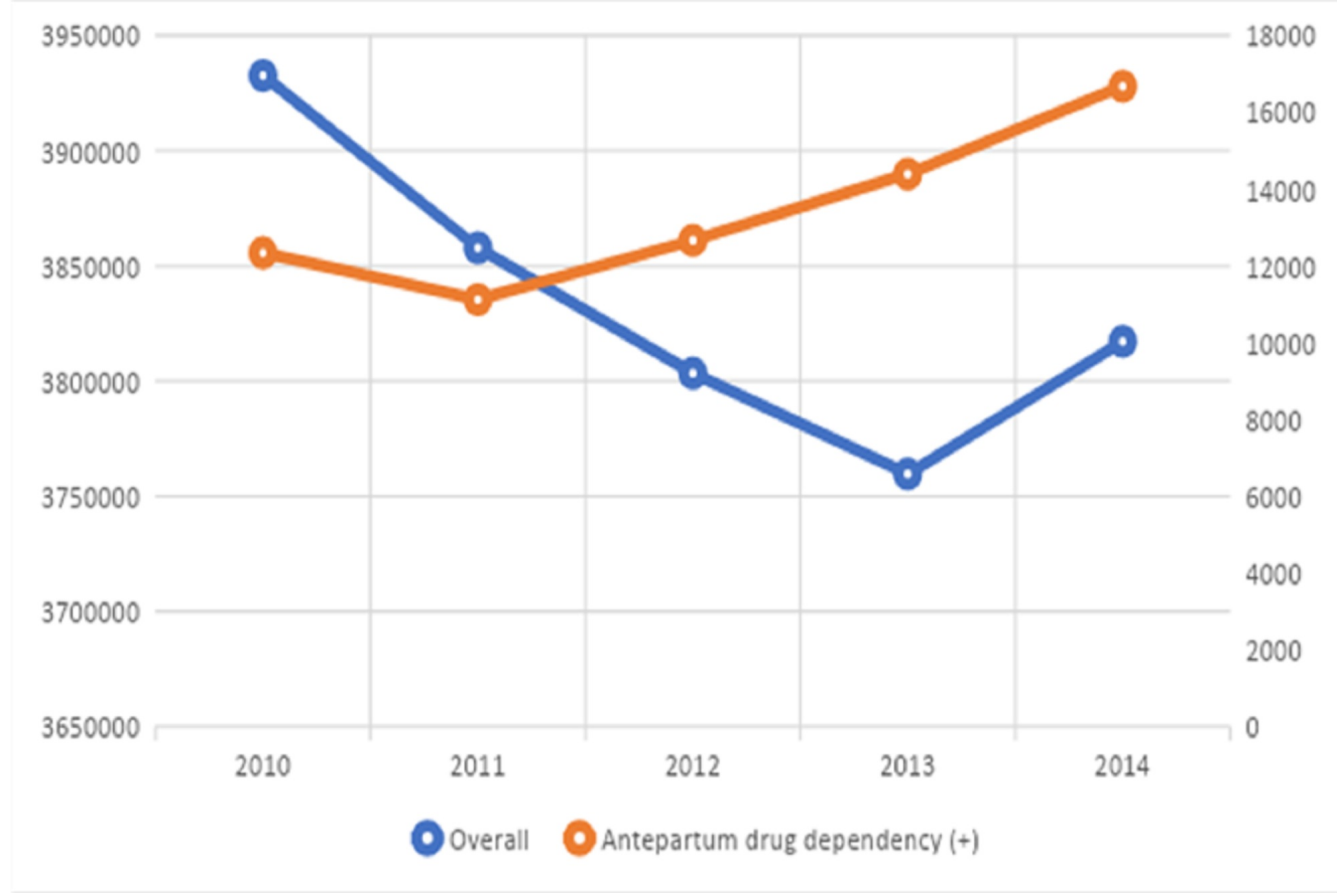
Trends of hospitalizations for pregnancy- or birth-related complications, 2010 to 2014

We analyzed 19,170,561 hospitalizations for principal discharge diagnosis of pregnancy- or birth-related complications, of which 67,180 patients (0.35%) had co-diagnosis of drug dependency in pregnancy/antepartum. There was an initial decline in ADD inpatients from 2010 to 2011 followed by a steady increase in the trend with an increase of 50% from 2011 (n = 11,118) to 2014 (n = 16,675) as shown in Figure 1.

Demographic characteristics

Patients aged 21-30 years represented a higher proportion of inpatients with pregnancy- or birth-related complications (64.2%) and had 1.7-fold higher odds [95% confidence interval (CI): 1.61-1.75] for ADD. White pregnant females (77.5%), and those from low-income families (below the 25th percentile, 37.1 %) constituted a higher proportion of the female pregnant inpatients with comorbid ADD.

Medical and psychiatric comorbidities

Among medical comorbidities, iron-deficiency anemias were most prevalent in pregnant inpatients (12.0% in ADD vs. 9.2% in non-ADD) followed by obesity and hypertension. In the adjusted regression model, none of the medical comorbidities had higher odds of association in pregnant inpatients with ADD compared to the non-ADD group.

Depression was the most prevalent psychiatric comorbidity in ADD inpatients (12.9%) with two-fold higher odds (95% CI: 1.98-2.13) than non-ADD inpatients. Also, ADD inpatients had three-fold higher odds (95% CI: 2.83-3.11) for comorbid psychosis compared to non-ADD inpatients.

Among patients with SUD, the opioid-use disorder was most prevalent in pregnant inpatients with ADD (67.3%), and it showed 487-fold higher odds (95% CI: 476.59-498.46) compared to non-ADD inpatients. The second most prevalent substance abused was cannabis (11.2%) and ADD inpatients had four-fold higher odds (95% CI: 3.97-4.35) for cannabis use disorder than non-ADD inpatients. Other SUD that was significantly associated with ADD inpatients were related to cocaine (5.7%, increased by 1.5 times), amphetamine (4.0%, increased by 2.5 times), and alcohol (2.4%, increased by 3.7 times) as shown in Table 1.

**Table 1:** Sociodemographic characteristics and comorbidities in pregnant inpatients with antepartum drug dependence OR: odds ratio; CI: confidence interval

Variable	Antepartum drug dependency		Logistic regression model		
	No, %	Yes, %	OR	95% CI	P-value
Age, years					
12–20	11.9	5.5	Reference		
21–30	53.7	64.2	1.68	1.61–1.75	<0.001
31–40	34.4	30.3	1.54	1.47–1.61	<0.001
Race					
White	52.5	77.5	Reference		
Black	15.5	10.2	0.59	0.58–0.62	<0.001
Hispanic	21.2	8.5	0.52	0.50–0.54	<0.001
Other	10.8	3.9	0.46	0.44–0.48	<0.001
Median household income, percentile					
0–25^th^	28.1	37.0	Reference		
26^th ^–50^th^	25.1	27.1	0.95	0.92–0.97	<0.001
51^st^ –75^th^	25.1	22.5	0.86	0.84–0.89	<0.001
76^th ^100^th^	21.8	13.4	0.73	0.71–0.75	<0.001
Severity of illness, loss of functions					
Minor	60.2	14.5	Reference		
Moderate	32.6	54.3	3.77	3.67–8.88	<0.001
Major	7.2	31.2	7.35	7.11–7.59	<0.001
Psychiatric comorbidities					
None	-	-	Reference		
Depression	2.2	12.9	2.06	1.98–2.13	<0.001
Psychosis	0.9	9.6	2.97	2.83–3.11	<0.001
Substance use disorders					
None	-	-	Reference		
Alcohol	0.1	2.4	3.68	3.35–4.04	<0.001
Tobacco	0.2	1.2	1.10	0.99–1.23	0.091
Cannabis	0.7	11.2	4.16	3.97–4.35	<0.001
Opioid	0.2	67.3	487.40	476.59–498.46	<0.001
Cocaine	0.1	5.7	1.56	1.46–1.66	<0.001
Amphetamine	0.1	4.0	2.56	2.37–2.77	<0.001
Barbiturate	0	1.7	0.41	0.37–0.45	<0.001
Medical comorbidities					
None	-	-	Reference		
Deficiency anemias	9.2	12.0	0.97	0.94–1.01	0.105
Diabetes	1.2	1.6	0.77	0.70–0.83	<0.001
Hypertension	2.4	4.3	0.77	0.73–0.82	<0.001
Hypothyroidism	2.6	2.5	0.88	0.82–0.94	<0.001
Obesity	5.9	6.4	0.83	0.79–0.87	<0.001

Hospitalization outcomes 

About half of the pregnant inpatients with ADD had moderate severity of illness due to pregnancy- or birth-related complications, with a four-fold higher odds for the same (95% CI: 3.67-8.88) compared to the non-ADD inpatients. Also, 31.2 % of ADD inpatients had a seven-fold higher odds of association (95% CI: 7.11-7.59) for major severity of illness.

Pregnant inpatients with ADD had a higher LOS with a mean difference of 0.88 days (95% CI: 0.904-0.865) and incurred higher total charges by 3,797 USD (95% CI: 3,927-3,666) per inpatient admission for pregnancy- or birth-related complications compared to non-ADD inpatients as shown in Table 2.

**Table 2:** Difference in length of stay and charges due to antepartum drug dependence MD: mean difference; CI: confidence interval *Figures in parentheses represent standard deviation

Variable	Antepartum drug dependency		T-test for equality of means		
	No	Yes	MD	95% CI	P-value
Mean length of stay, days	2.69 (2.55)*	3.57 (4.81)*	0.88	0.904–0.865	<0.001
Mean total charges, USD	15,461 (17,003)*	19,258 (28,525)*	3,797	3,927–3,666	<0.001

## Discussion

Pregnant women between the ages of 21 to 30 and 31 to 40 years had a higher likelihood of ADD by 1.5 to 1.7 times compared to adolescents. This could possibly be due to various biopsychosocial factors including overcrowded localities with violence and illness, homelessness, and sexually transmitted diseases, which render them more vulnerable to addiction [14]. Globally, 41% of all pregnancies are unintended, part of which may be due to substance abuse [15], which is often under-reported in pregnant women [16,17]. Also, shame, embarrassment, and fear of harm to the fetus may prevent pregnant women from seeking help [18].

White women had higher rates of tobacco, alcohol, and illicit substance use compared to Blacks and Hispanics, which may have been primarily due to differences in self-reporting of substance use [19]. This was also seen in our study as about three-fourth of the pregnant women with ADD were White. Nativity also plays an important role in determining the type of substance used as alcohol use was four times higher than tobacco among immigrant women, whereas American-born women showed a different pattern [20]. Newborns of White mothers were equally at risk of alcohol and tobacco exposure, while newborns of Black mothers were mostly affected by illicit substances [20].

Greater number of pregnant women from lower socioeconomic status (SES) abuse illicit substances due to certain problems like intimate partner violence, living in unsafe neighborhoods, multiple dependents, and widespread accessibility to illicit substances [21,22]. In our study, we found an inverse relationship between the prevalence of comorbid ADD and median household income. Addiction itself greatly affects one`s productivity, perpetuating a vicious cycle of poverty and substance use. Lack of awareness about substance use and its harm to the mother and fetus plays a role in the widespread use of substances in women from low SES [21,22].

In our study, pregnant women with ADD had 487 times higher risk of opioid abuse. Maeda et al. reviewed the outcomes in 57 million pregnancy and deliveries from 1998 to 2011 and found that 113,105 (0.2%) had comorbid opioid-use disorder [23]. The prevalence increased by 127% from 1.7 per 1,000 deliveries in 1998 to 3.9 per 1,000 deliveries in 2011. Opioid abuse/dependence during pregnancy increased the odds of major obstetrical morbidity and mortality and in-hospital mortality [23]. Next, we found cannabis abuse was as high as four times compared to other illicit substances. Cannabis produces biphasic effects. At a low dose, cannabis is sympathomimetic, causing tachycardia, and at a higher dose, it is parasympathomimetic, causing hypotension and bradycardia [24]. Cannabinoid hyperemesis syndrome and/or cannabis withdrawal with or without psychosis may result in abortion or preterm labor [24].

Comorbid stimulant abuse in pregnant women in our inpatient sample had about 1.5 to 2.5 times higher risk. Cocaine causes prolonged adrenergic stimulation, increased vasoconstriction of the blood vessels, and tachyarrhythmias, leading to abruptio placenta, fetal hypoxia, and fetal distress syndrome [25,26]. Amphetamine stimulates the release of catecholamines from presynaptic vesicles, resulting in hypertension, arrhythmia, and seizures, which lead to premature delivery and abortion [27].

A nationwide study found that antepartum mental disorders are associated with increased LOS and higher inpatient charges by USD 1,889 [8]. Similarly, we found that pregnant inpatients with ADD had an extended LOS and higher total charges by USD 3,797. So, it is important to build an integrated clinical care model to reduce the higher healthcare economic burden and pregnancy- or delivery-related complications by systematic screening for SUD. Pregnant mothers with SUD should be educated regarding its adverse effects in the antepartum period, which may increase their personal motivation. A brief therapy extending from two to six sessions of cognitive-behavioral therapy or motivational enhancement therapy is a useful intervention in counteracting ADD. Lastly, it is vital to bridge the gap between community programs and early intervention and referral activities.

## Conclusions

Women with ADD hospitalized for pregnancy- or birth-related complications were generally young (21-30 years) Whites and those from low SES. Opioid abuse was the most prevalent substance abuse during pregnancy followed by abuse of cannabis and stimulants (cocaine/amphetamine). ADD worsens the severity of pregnancy- or birth-related complications and increases LOS by 0.8 day and total inpatient charges by USD 3,797. These factors result in a significant increase in healthcare burden. It is important to integrate SUD services with primary and maternal healthcare in order to improve pregnancy-related hospitalization outcomes.
